# Sydenham's chorea in a 16‐year‐old female from Bhutan: A case report

**DOI:** 10.1002/ccr3.9047

**Published:** 2024-06-21

**Authors:** Tshering Penjor, Thinley Dorji, Sangay Wangchuk

**Affiliations:** ^1^ Department of Internal Medicine Central Regional Referral Hospital Gelephu Bhutan

**Keywords:** acute rheumatic fever, diagnostic criteria, rheumatic heart disease, subclinical carditis, Sydenham's chorea

## Abstract

**Key Clinical Message:**

Rheumatic heart disease is a preventable disease. Patients may not present with a typical history of sore throat and polyarthritis but may present with Sydenham's chorea. We should not rely completely on clinical findings to rule out carditis. Echocardiography should be done to rule out subclinical carditis.

**Abstract:**

Sydenham's chorea is a major manifestation of rheumatic fever. It occurs primarily in children and is seen rarely after the age of 20 years. We describe a 16‐year‐old girl who presented with purposeless involuntary movements of her upper and lower limbs. Laboratory blood reports showed raised erythrocyte sedimentation rate and anti‐streptolysin O. 2D Doppler Echocardiography confirmed subclinical carditis, thickened mitral and aortic valve with mild mitral regurgitation. She was managed as Acute Rheumatic Fever with oral Phenoxymethyl penicillin and Carbamazepine. At the latest follow‐up interviewing the caregiver, the patient had no sequelae. Early diagnosis is key to preventing late consequences of acute rheumatic fever and rheumatic heart disease. Sydenham's chorea is a rare presentation of acute rheumatic fever. The absence of clinical carditis does not rule out carditis.

## 1 INTRODUCTION

Sydenham chorea represents the most common cause of acquired chorea in childhood.^1^ It is one of the major criteria for the diagnosis of acute rheumatic fever.^2^ In Sydenham chorea, there are both neurological abnormalities and psychiatric disorders. The neurological abnormalities comprise involuntary choreatic movements, incoordination of voluntary movements, muscular weakness and hypotonia.^3^ Psychiatric disorders include emotional lability, hyperactivity, distractibility, obsessions, and compulsions.^2^, ^3^ Choreatic movements are involuntary, irregular, purposeless, non‐rhythmic, abrupt, rapid, and unsustained. It disappears in sleep.^2^

The most common age for the onset of acute rheumatic fever is from 5 to 15 years old.^1^ Rheumatic fever is the major cause of acquired heart disease in children and up to 60% of people who present with Sydenham chorea develop rheumatic heart disease.^2^ Rheumatic fever is an acute non‐suppurative inflammatory complication of group A streptococcal pharyngitis.^4^ Depending on genetic predisposition and the virulence of the infecting strain, 0.3%–3% of people with GAS pharyngitis develop acute rheumatic fever.^4^

Globally, some 30 million people are currently thought to be affected by rheumatic heart disease, and in 2015 rheumatic heart disease was estimated to have been responsible for 305,000 deaths and 11.5 million disability‐adjusted life years lost. The worst affected are the African, Southeast Asia and the Western Pacific regions, accounting for 84% of all prevalent cases and 80% of all estimated deaths due to rheumatic heart disease in 2015. India has the highest global prevalence, with about 27% of all cases globally.^5^ The estimated average prevalence in India is 0.5/1000 children in age group of 5–15 years.^6^ Bhutan, in Southeast Asia region, also has a high burden of cases but due to the unavailability of data, it is difficult to say how high. In this article, we present a case of Sydenham's chorea leading to the diagnosis of  acute rheumatic fever in a female teenager in Bhutan.

Sydenham's chorea is a neuropsychiatric disorder that is mediated by anti‐neuronal antibody.^1^ Following group A beta‐hemolytic streptococcus infection, the antibodies which arise in response, cross react with epitopes on neurons within the basal ganglia, frontal cortex, and other regions. The autoimmune process causes hyperemia, endothelial swelling, and perivascular round‐cell infiltration which result in dopaminergic dysfunction.^1^

## 2 CASE HISTORY/EXAMINATION

A 16‐year‐old girl presented to the outpatient department at a regional referral hospital in Bhutan with involuntary movements of both upper and lower limbs for 2 weeks duration. Her left‐sided extremities were more affected than the right side. The symptoms had affected her daily activities like brushing her teeth, eating, and writing, because of which she was unable to continue her school. Her parents did not recall any febrile illness, sore throat or any major trauma in the recent past. There was no history of joint pain or swelling, rashes or chest pain. No history of drug intake, over‐the‐counter medications or herbal medications. There were no similar problems in the past or any family history of similar problem. She had a normal menstrual cycle.

On examination, she was alert, well‐oriented in time, place and person. Pulse = 82/min, regular in rhythm, normal character and volume, BP = 108/61 mmHg in right arm, temperature = 98.4°F. There was no pallor, icterus, cyanosis, clubbing, oedema, skin rash, or lymphadenopathy.

Nervous system: Higher mental function was intact, with no cranial nerve deficit. Her speech was slow, with reduced verbal fluency. There were jerky involuntary movements of her left‐sided extremities with writhing movements of her fingers. The muscle tone, deep tendon reflexes and muscle power were symmetrical and normal. There were no cerebellar signs. Gait was unsteady with episodic jerky movements of limbs. Cardiovascular system: No chest wall deformities, first and second heart sounds were heard normal with no murmurs or rub. Respiratory system: bilateral vesicular breaths. Abdomen: Soft with no palpable liver or spleen.

## 3 METHODS

### 3.1 Investigations and treatment

**FIGURE 1:**
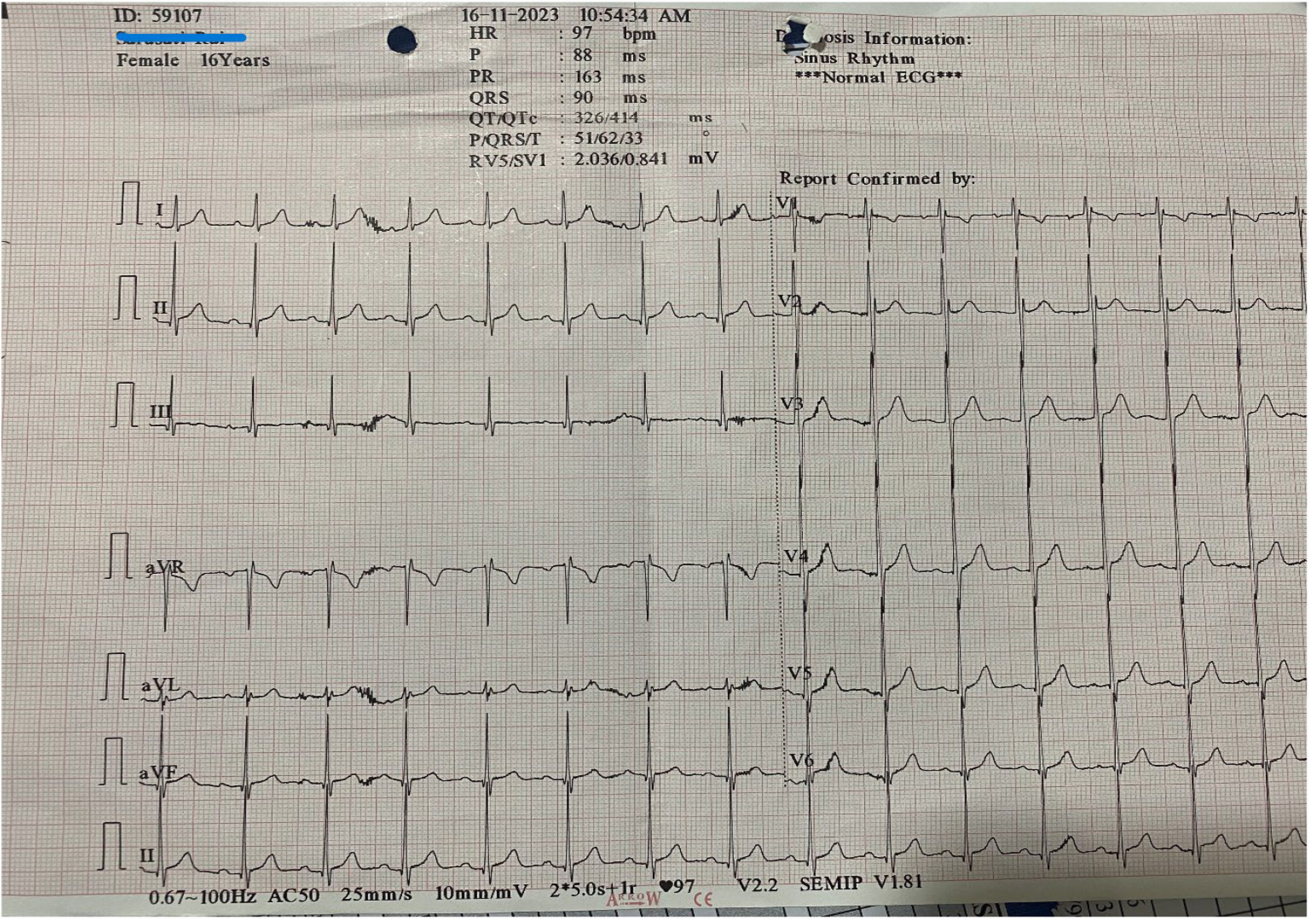
Electrocardiogram of 16‐year‐old female with Sydenham's chorea and subclinical carditis showing normal sinus rhythm.

Complete blood count, renal parameters, liver enzymes, and electrolytes were unremarkable (Table 1). Erythrocyte sedimentation rate was raised, 68 mm/h, Anti‐streptolysin O titre was 245 IU/mL. Electrocardiogram: Normal sinus rhythm (Figure 1). Echocardiography showed thickened mitral and aortic valves with mild mitral regurgitation with normal ejection fraction. C‐reactive protein, thyroid‐stimulating hormone level, autoimmune panels and MRI brain were not done because of its unavailability at our centre.

**TABLE 1:** Summary of investigation findings in a 16‐year‐old female with Sydenham's chorea treated at the Central Regional Referral Hospital, Bhutan, 2023.

Test parameters	Patient's value	Normal range
Hemoglobin (g/dL)	12.3	11.0–16
Mean corpuscular volume (fL)	79	73–88
White blood cell count (/μL)	6270	5000–15,000
Neutrophil (%)	60	35–55
Platelet (/μL)	458,000	150,000–450,000
Urea (mg/dL)	12	15–45
Creatinine (mg/dL)	1.0	0.4–1.3
Alanine aminotransferase (IU/L)	14	5–40
Aspartate aminotransferase (IU/L)	23	5–40
Bilirubin, total (mg/dL)	0.6	0.1–1.2
Bilirubin, direct (mg/dL)	0.1	<0.2
Estimated sedimentation rate (mm/h)	68	0–15
Anti‐streptolysin O (IU/mL)	245	<150
Electrocardiogram	Normal sinus rhythm	
2D echocardiography	Thickened mitral and aortic valve with mild mitral regurgitation. Ejection fraction 60%	

A diagnosis of Sydenham chorea was made based on the clinical probability and was managed with oral Phenoxymethylpenicillin 500 mg twice a day and Carbamazepine 200 mg twice a day.

## 4 RESULTS

With treatment, her choreatic movements reduced; she was able to carry out her daily activities independently. She was discharged and is currently doing well on follow up. She has been started on secondary prophylaxis with oral Phenoxymethylpenicillin 250 mg twice a day instead of intramuscular injection of Benzathine penicillin G as preferred by the patient and her parents.

## 5 DISCUSSION

Our case provides evidence of the persistence of acute rheumatic fever in Bhutan where clinical data and publication are very limited. In contrast to carditis and arthritis, which typically present within 21 days, the onset of Sydenham chorea usually occurs 1–8 months after the inciting infection.^2^ By the time the patient presents to medical care, they might not remember the inciting infection and therefore we cannot solely depend on history to make a diagnosis. Alternative aetiologies of acquired chorea such as autoimmune or inflammatory, cerebrovascular, drugs, infections, metabolic disorders, or neoplasia should be kept in mind.^2^

The diagnosis of rheumatic fever is based on the Jones criteria (Table 2). The most common manifestation which is present in 80% of patients is arthritis, described as painful, migratory and transient. Frequently affected joints are the knees and ankles. Carditis occurs in 40%–75%, erythema marginatum and subcutaneous nodules are rare, occurring in less than 10% of patients.^4^ Sydenham chorea is also a rare presentation, occurring in 10%–30%.^4^ In our case, the patient presented with Sydenham chorea which hints at a possible larger number of cases with acute rheumatic fever that may be underdiagnosed or under‐reported.

**TABLE 2:** Jones criteria for rheumatic fever (revised 2015) and patient's condition and symptoms.

Symptoms	Low‐risk population (ARF incidence ≤2 per 100,000 school‐aged children or all‐age RHD prevalence of ≤1 per 1000 population per year)	Moderate/high‐risk population (Children not clearly from low‐risk population)	Patient's case
Major criteria			
Carditis	Clinical and/or subclinical	Clinical and/or subclinical	Subclinical
Arthritis	Only polyarthritis	Monoarthritis, polyarthritis and or polyarthralgia	No
Chorea	Yes	Yes	Yes
Erythema marginatum	Yes	Yes	No
Subcutaneous nodules	Yes	Yes	No
Minor criteria			
Fever	≥38.5 °C	≥38 °C	No
Arthralgia	Polyarthralgia	Monoarthralgia	No
ESR	Peak ESR ≥60 mm in 1 hour	Peak ESR ≥30 mm in 1 hour	Yes
CRP	≥3.0 mg/dL	≥3.0 mg/dL	–
PR interval (duration depends on age, unless carditis is found)	Prolonged	Prolonged	No

Abbreviations: ARF, Acute rheumatic fever; CRP, C‐reactive protein; ESR, Erythrocyte sedimentation rate; RHD, Rheumatic heart disease.

Carditis can be diagnosed clinically in the presence of an audible murmur consistent with aortic or mitral regurgitation on auscultation.^2^ However, more recent studies on patients with acute rheumatic fever have brought out the shortcomings of auscultation in identifying valve diseases which do not result in hemodynamic abnormalities consisting of murmurs.^4^ This has resulted in the identification of subclinical carditis by echocardiography.^2^ In Bhutan, we have facility for echocardiography only in three tertiary hospitals out of 20 districts in the country leading to high chances of missing the diagnosis of subclinical carditis.

In our case, the patient had subclinical carditis in the form of mitral regurgitation diagnosed with echocardiography. The patient had two major criteria, Sydenham chorea and subclinical carditis, and minor criteria, raised ESR and ASO titre thereby fulfilling the Jones criteria for rheumatic fever (Table 2).

There were at least two cases reported with a similar presentation, the first case was 8‐year‐old Spanish boy. He was treated with prednisone 2 mg/kg/day for 2 weeks with gradual tapering for 6 weeks period and carbamazepine 17 mg/kg/day. He had complete resolution of movement disorder after 1 month.^2^ The second case was a 10‐year‐old girl who was treated with intravenous immunoglobulin (400 mg/kg/day) for 5 days, prednisone 2 mg/kg/day for 2 weeks with gradual tapering over 6 weeks. She recovered completely after 3 weeks.^2^ In our case, she was started on carbamazepine 200 mg twice a day following which her choreatic movements reduced significantly. After discharge from our centre, she could not come back for follow‐up, so we could not monitor her ESR and other laboratory parameters. However, on telephonic follow‐up, her father claims she is doing well.

Rheumatic heart disease is preventable. It is a serious public health problem, especially in low‐ and middle‐income countries with limited capacities for the diagnosis and timely management of streptococcal infection.^5^ It exerts massive economic effects globally, mainly because of premature death in children and working‐age adults.^7^ Globally, acute rheumatic fever and rheumatic heart diseases are seen in developing nations or among disadvantaged populations within developed nations.^8^ The global cost of deaths due to rheumatic heart disease in 2010 was estimated to be US$ 2200 billion (discounted) or US$ 5400 billion (undiscounted).^5^ The most devastating effects are on children and young adults in their most productive years because it leads to increased school absenteeism and dropout, and lost wages.^5^ The patient, in our case, dropped out of school, but will be continuing in the next academic session after the control of chorea.

The prevention, control and elimination of rheumatic heart disease is increasingly being recognized as an important developmental issue by the World Health Organization.^5^ The barriers to prevention, control and elimination of rheumatic heart disease are poor primary and secondary prevention and access to primary health care, inadequate numbers and training of health workers at all levels, the neglect of rheumatic fever and rheumatic heart disease in national health policies and budgets, the paucity of data to enable targeting of prevention efforts, limited understanding of rheumatic fever and/or rheumatic heart disease in affected communities, and inaction on the social determinants of the disease and inequities in health.^5^ In Bhutan, we have communities where people prefer help from local healers than to visit health centres. In our case, the patient's father initially refused our help and wanted to go to a local healer but after explaining the disease condition to him, he agreed and remained with us.

## 6 CONCLUSION

Acute rheumatic fever continues to be a major health burden in our country. New‐onset chorea in childhood should raise the suspicion of Sydenham's chorea. Echocardiography should be done to diagnose subclinical carditis rather than depending only on clinical findings. Early diagnosis and management of acute rheumatic fever is crucial in preventing its recurrence and progressive damage to heart valves.

## AUTHOR CONTRIBUTIONS

**Tshering Penjor:** Conceptualization; formal analysis; investigation; project administration; resources; supervision; validation; visualization; writing – original draft; writing – review and editing. **Thinley Dorji:** Conceptualization; investigation; project administration; resources; supervision; validation; visualization; writing – review and editing. **Sangay Wangchuk:** Conceptualization; investigation; validation; visualization; writing – review and editing.

## FUNDING INFORMATION

There are no funders to report for this submission.

## CONFLICT OF INTEREST STATEMENT

There is no any conflict of interest.

## CONSENT

Written informed consent was obtained from the patient to publish this report in accordance with the journal's patient consent policy.

## Data Availability

Data sharing not applicable: no new data generated.
